# Thioredoxin 1 as a serum marker for breast cancer and its use in combination with CEA or CA15-3 for improving the sensitivity of breast cancer diagnoses

**DOI:** 10.1186/1756-0500-7-7

**Published:** 2014-01-06

**Authors:** Byung-Joon Park, Mee-Kyung Cha, Il-Han Kim

**Affiliations:** 1 Department of Life Sciences & Technology, Paichai University, Daeduk Campus, Techno 1Ro, Daejeon, Republic of Korea

**Keywords:** Breast cancer, Diagnosis, Thioredoxin 1, Companion marker, Carcinoembryonic antigen (CEA), Cancer antigen 15–3 (CA15-3), Reactive oxygen species

## Abstract

**Background:**

The human cytosolic thioredoxin (Trx) contains a redox-active dithiol moiety in its conserved active-site sequence. Activation by a wide variety of stimuli leads to secretion of this cytoplasmic protein. Function of Trx1 has been implicated in regulating cell proliferation, differentiation, and apoptosis. The aim of this study was to assess the clinical significance of serum Trx1 level in patients with breast carcinoma.

**Results:**

To clarify whether serum levels of Trx1 could be a serum marker for breast carcinoma, we measured the serum levels of Trx1 in patients with various carcinomas (breast, lung, colorectal, and kidney cancers) using an ELISA, and investigated its associations with the tumour grading from I to III. At the cut-off point 33.1725 ng/ml on the receiver operating characteristic curve (ROC) Trx1 could well discriminate breast carcinoma from normal controls with a sensitivity of 89.8%, specificity 78.0%, and area under the ROC (AUC) 0.901 ± 0.0252. The serum level was well correlated with the progress of the breast carcinoma. We also investigated the diagnostic capacity of CEA and CA15-3 for the early detection of metastatic breast cancer comparing that of Trx1. In contrast to the serum CEA and CA15-3 tumour markers, the serum Trx1 levels of the early cancer (grade I) patients were significantly higher than those of normal control subjects, showing a high diagnostic sensitivity and selectivity (89.4% sensitivity, and 72.0% specificity). The serum levels of Trx1 in various patients with lung, colorectal, and kidney carcinomas indicate that the level of Trx1 is significantly higher than those of other cancer patients. Combinational analysis of CEA or CA15-3 with Trx1 for the detection of breast cancer suggest that the diagnostic capacity of CEA or CA15-3 alone for the early detection of breast cancer, especially regarding sensitivity, is significantly improved by its combination with Trx1.

**Conclusions:**

Taken together, we conclude that serum Trx1 is useful for the early diagnosis of breast cancer or the early prediction prognosis of breast cancer, and therefore has a valuable use as a diagnostic marker and companion marker to CEA and CA15-3 for breast cancer.

## Background

Organisms living under aerobic conditions are exposed to reactive oxygen species (ROS) such as superoxide anion (O_2_^–^), hydrogen peroxide (H_2_O_2_), and nitric oxide (NO), which are generated by redox metabolism, mainly in mitochondria. It has been demonstrated *in vitro* that ROS in small amounts participate in many physiological processes such as signal transduction, cell differentiation, apoptosis, and modulation of transcription factors [1-4]. All organisms, from prokaryotes to primates, are equipped with different defensive systems to combat the toxic processes of ROS. Regulation mechanisms of ROS play a crucial role in tumour development. Transformed cells are known to generate more ROS than normal cells [5,6]. ROS not only contribute to tumor progression by amplifying genomic instability but also transformed cells use ROS signals to sustain proliferation [5].

Thioredoxin (Trx) is a 12-kDa oxidoreductase that is kept in the reduced state by thioredoxin reductase in a NADPH-dependent reaction. Serving as a general disulfide oxidoreductase, thioredoxin facilitates the reduction of other proteins by a redox mechanism based on reversible reduction of a disulfide to two cysteine thiol groups, thereby recovering the normal function of the proteins. Trx1 as an antioxidant protein is induced by various kinds of oxidative stresses [7-10]. In mammalian cells, Trx1 is also involved in the regulation of ROS levels and thus in cell death. In addition to its critical role in the regulation of cellular redox homeostasis, Trx1 has multiple actions in the cell. Therefore, Trx1 is potentially important in conjunction with the onset of many diseases including inflammatory diseases, heart failure, cancer, etc. Trx1 plays an important role in regulating cancer cell growth, for example, by modulating the DNA binding activity of transcription factors, including nuclear factor-κB, p53, and glucocorticoid and estrogen receptors [11-16]. Immunohistochemical analysis with anti-Trx1 antibodies revealed the expression of Trx1 in cancer cells in various tissues such as the liver, colon, pancreas, and the uterine cervix, indicating the implication of Trx1 in oncogenesis [17-19].

Breast cancer is reported to occur at a higher rate in women of advanced countries than do other cancers [20]. The incidence of breast cancer and the mortality from breast cancer in low-developed countries are expected to increase for a significant period of time in the future in consideration of the trend of because of the westernization of living. As they grow, breast cancer cells, like other cancer cells, generally infiltrate adjacent tissues or metastasize into the lymph nodes. In most breast cancer cases, the patients did not have many detectable symptoms or they did not examine their breasts by themselves. Therefore, it is very important to effectively diagnose early breast cancer to reduce mortality from breast cancer. To decrease the mortality from breast cancer, early diagnosis is the most important thing. It is also important to do an adjuvant therapy that is suitable in light of the prognosis after the primary initial treatment. For the diagnosis of breast cancer, various methods are used in combination. To supplement such breast screening methods, attempts have been made to use blood tumor marker levels to diagnose breast cancer. Although studied for their values as diagnosis or prognosis factors, the application of conventional tumor markers is accompanied by limitations, and there are no officially recommended breast cancer markers.

There has been a need for a diagnostic marker and a method for breast cancer that allows the accurate diagnosis of early breast cancer and allows the prognosis of breast cancer to be made. Almost few studies have been done on the use of Trx1 as a diagnostic marker for breast cancer. Recently, we reported that Trx1 are overexpressed in human breast carcinoma tissues and the expression levels are associated with tumor grade [21]. Found to be overexpressed in human breast cancer tissues, Trx1 allows the diagnosis and prognosis of breast cancer and thus is useful as a diagnostic marker for breast cancer. The striking induction of Trx1 in breast cancer tissues may enable their use as a blood diagnostic marker for breast cancer.

Intensive and thorough research into the simple and selective diagnosis of breast cancer using blood, which is a relatively easily obtainable specimen, resulted in the finding that healthy persons and patients affected with breast cancer exhibit different plasma levels of Trx1. Trx1 can be used as a diagnostic marker for breast cancer, which is characterized by a remarkable, highly specific, and sensitive elevation of the serum Trx level.

## Methods

### Subjects

All clinical grade sera of normal persons (control) and various cancer patients were obtained from two global supplier of human samples, Asterand (http://www.asterand.com) and Bioserve (http://www.bioserve.com). All the sera and the clinical information were provided from the global supplier as summarized in Table 1. All samples were collected from their collaborating clinical sites with full adherence to proper informed consent, as well as their strict institutional review board (IRB) and Health Insurance Portability and Accountability Act (HIPAA) compliance. To make them suitable for a biomarker study, all the sera were collected and treated according to the instructions of the FDA (Food and Drug Administration) and the NCI (National Cancer Institute).

**Table 1:** Clinical information of serum of normal control and patients with various cancers

**Breast carcinoma**		**Non-small Cell Lung Cancer (NSCLC)**		**Colorectal carcinoma**		**Kidney carcinoma**		**Normal control**	
**Characteristics**		**Characteristics**		**Characteristics**		**Characteristics**		**Characteristics**	
No. of samples	197	No. of samples	111	No. of samples	64 (Rectal 14)	No. of samples	30	No. of samples	100
Female	197	Male	50	Male	33 (Rectal 7)	Male	17	Male	50
Mean age (years)	48.54 ± 18.43 (20–91)	Female	61	Female	31 (Rectal 7)	Female	13	Female	50
Stage I	66 (35:31)*	Mean age (years)	41 ± 10.39 (41–85)	Mean age (years)	63.84 ± 12.20 (39–78)	Mean age (years)	55.07 ± 11.22 (34–76)	Male mean age (years)	44.54 ± 14.85 (20–76)
Stage II	70 (37:33)	Stage I	39 (IA 19, IB 20)	Stage I	6	Stage I	26	Female mean age (years)	43.70 ± 14.95 (18–71)
Stage III	61 (34–27)	Stage II	32 (IIA 16, IIB 16)	Stage II	36 (Rectal 7)	Stage II	4	Female/Male	44.12 ± 14.83
Lobular type	106	Stage III	30 (IIIA 21, IIIB 90)	Stage III	22 (Rectal 7)			Mean age (years)	(18–76)
Ductal type	91	Stage IV	10						

All sera obtained from Caucasian White.

*First and second values are # of lobular and ductal carcinoma breast cancer.

### Assay for Trx1 protein

An indirect ELISA (Enzyme-linked immunosorbent assay) was performed to quantitatively analyze the level of serum Trx1 protein. Serum obtained after the centrifugation of blood samples taken from normal persons and breast cancer patients were used for quantitative protein analysis with an indirect ELISA kit using antibodies of Trx1 (Express ELISA kit for rabbit, GenScript). In this regard, rabbit polyclonal antibodies to respective antigens were obtained by injecting purified human Trx1 into rabbits to form antisera and purifying the antisera on a Protein A column. After the pre-coated antibody in a 96-well plate is allowed to react with the antigen, the antigen-antibody complex is treated with the secondary antibody conjugate, followed by immobilizing and washing 3 times with the washing solution. TMB (3,3′,5,5′-tetramethyl benzidine) was used as the substrate, and a sulfuric acid solution (2 M sulfuric acid) was used to stop the enzymatic reaction. The sandwich ELISA kits for CEA and CA15-3 from Abcom (Cambridge, MA, USA), were used to measure serum CEA and CA15-3 protein levels per the manufacturer’s instructions.

A standard curve was made from the absorbance at 450 nm of various concentrations of each protein. Serum protein levels were determined with reference to the standard curve from the mean values of three measurements of absorbance at 450 nm.

### Statistical analysis

For statistical analysis, the GraphPad Prism software (ver. 5.04) and MedCal statistical software (ver. 12. 4. 0. 0) were used for statistical analysis. We used the Pearson correlation to test for associations between different variables. The *t* test and one-way ANOVA were performed to calculate the P value. The P values were considered statistically significant if P < 0.05.

## Results

### Measurement of Serum Trx1 level in normal persons

**Figure 1:**
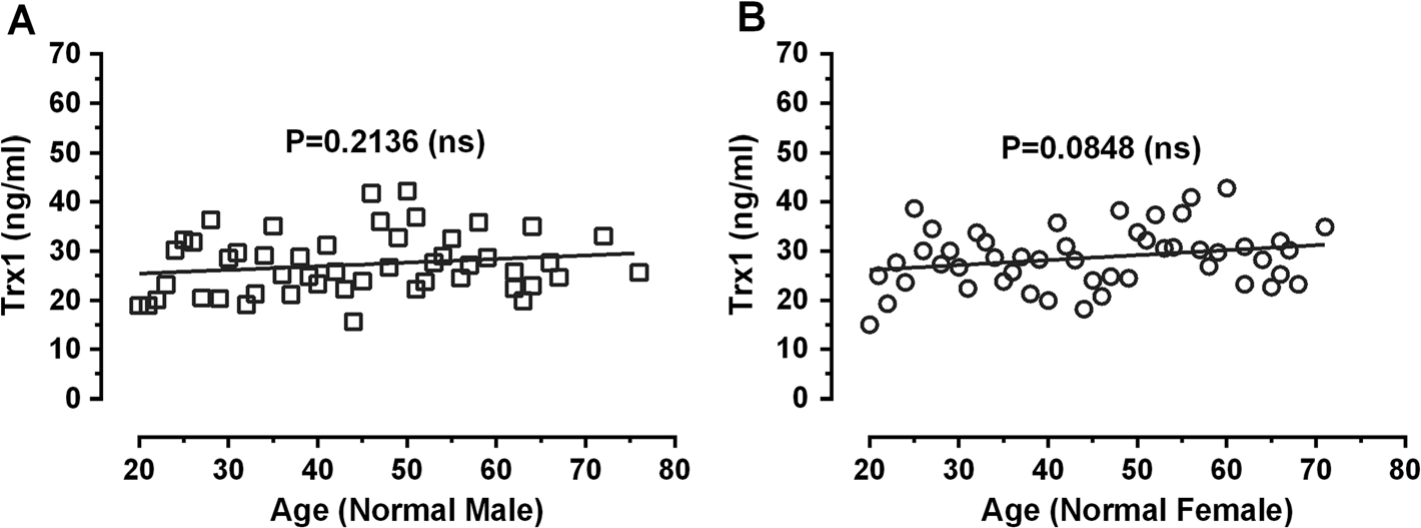
**Serum levels of Trx1 in normal male and female controls as a function of age.** Clinical information for each healthy person was provided by the supplier. The individual values of male **(A)** and female **(B)** normal controls are the average values of three measurements.

The serum levels of Trx1 in different normal individuals were analyzed by an ELISA. The measurements were performed on the sera of 50 normal female and male persons over a wide range of ages (20 – 76 for male, 18 – 71 for female) with a uniform distribution (44.54 ± 14.85 for male, 43.70 ± 14.95 for male, and 44.12 ± 14.83 for female/male; mean ± SD) (Table 1). The individual data were depicted as a function of age in Figure 1 and the statistics were summarized in Table 2. As shown in Table 2, the mean value of serum Trx1 levels were detected at 27.24 ± 6.155, 28.62 ± 6.054, 27.93 ± 6.113 (SD) ng/mL for male (NM), female (NF), and female/male (NFM), respectively. The plot shown Figure 1 indicates that the serum levels appear to be slightly elevated as the function of age for male (Figure 1A), and female (Figure 1B) normal controls, but they showed no statistical significance (P = 0.2136 for male controls, P = 0.0848 for female controls).

**Table 2:** Statistics for serum Trx1 levels of normal controls and various cancer patients

	**Normal (N) Control**				**Breast Cancer (BC)**					LC* (I-IV)	**CRC (I-III)**	**KC (I/II)**
	**NF**	**NM**	**NFM**	**I-III**	**I**	**II**	**III**	**L**	**D**			
**# of values**	**50**	**50**	**100**	**197**	**66**	**70**	**61**	**106**	**91**	**111**	**63**	**30**
**Minimum**	14.99	15.61	14.99	26.19	26.19	28.51	28.45	26.19	29.35	13.22	14.77	28.51
**25% Percentile**	23.98	22.35	23.26	35.53	32.99	35.9	39.45	35.82	34.91	25.23	27.71	25.79
**Median**	**28.5**	**26.22**	**27.64**	**38.98**	**36**	**38.97**	**43.98**	**39.03**	**38.98**	**31.89**	**31.09**	**30.96**
**75% Percentile**	32.04	31.97	31.96	44.77	39.08	44.74	50.45	44.72	44.94	37.84	38.29	35.54
**Maximum**	42.78	42.22	42.78	60.01	48.07	53.37	60.01	56.64	60.01	50	55.02	46.11
**Mean**	**28.62**	**27.24**	**27.93**	**40.12**	**35.93**	**40.1**	**44.67**	**40.03**	**40.22**	**31.71**	**32.92**	**31.12**
**Std. Deviation**	6.054	6.155	6.113	6.816	4.346	6.15	6.882	6.793	6.878	8.254	7.864	7.541
**Std. Error**	0.8561	0.8705	0.6113	0.4856	0.5349	0.7351	0.8812	0.6598	0.721	0.7835	0.9908	1.377
**Lower 95% CI**	26.9	25.49	26.72	39.16	34.86	38.63	42.91	38.72	38.79	30.16	30.94	28.31
**Upper 95% CI**	30.34	28.99	29.14	41.08	36.99	41.57	46.43	41.34	41.65	33.26	34.9	33.94

*Abbreviation used: F; Female, M; Male, BC; Breast Cancer, L; lobular carcinoma of breast cancer, D; ductal carcinoma of breast cancer, LC; lung cancer, CRC; colorectal cancer, KC; kidney cancer, CI; confidential interval.

### Measurement of serum Trx1 level in patients with breast cancer and other cancers

**Figure 2:**
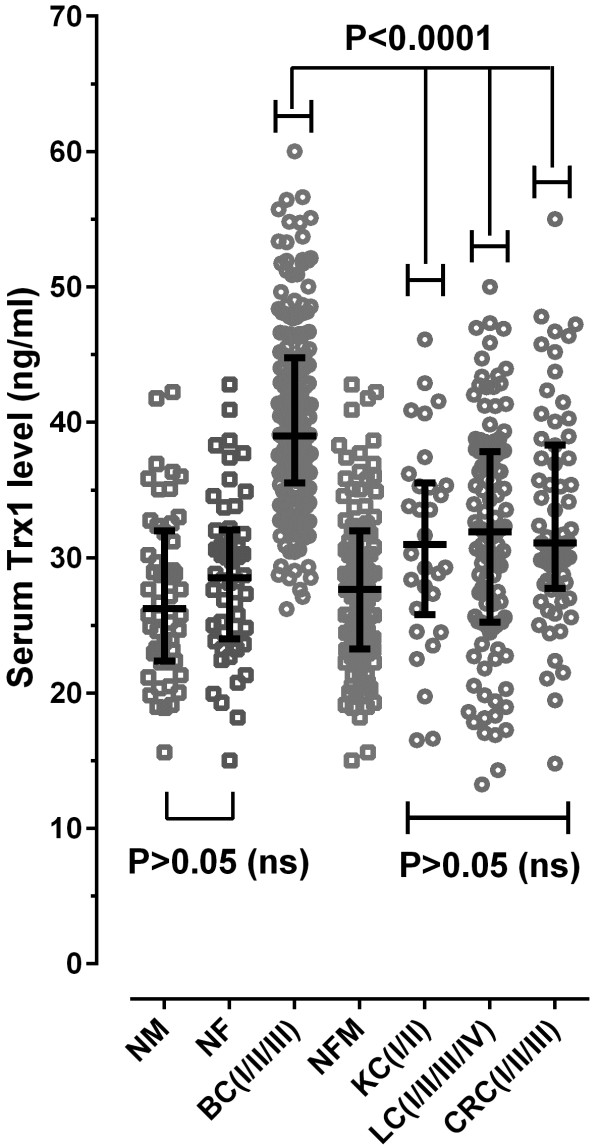
**Serum Trx1 levels in the breast cancer group and other cancer groups (kidney cancer, lung cancer and colorectal cancer).** Clinicopathological information for each patient was provided by the supplier. The individual mean value (n = 3) was depicted as a scatter plot. The median value of each group is depicted by horizontal lines, and the interquartile range is displayed by vertical lines extending to the up and down of the median. *Abbreviations*: BC, breast cancer; KC, kidney cancer; LC, lung cancer; CRC, colorectal cancer; NM, male normal control; NF, female normal control; NFM, female and male normal controls.

Trx1 levels in serum samples obtained from patients with breast cancer (BC), lung cancer (LC), kidney cancer (KC) and colorectal cancer (CRC) were assayed using ELISA, and the results were displayed as a scatter dot plot (Figure 2). The statistics were summarized in Table 2.

As can be seen in Figure 2, serum Trx1 levels were higher in the breast cancer group than in other cancer patients as well as in the female normal control (NF) and male normal control (NM), with statistical significance. Further, as shown in Table 2, the blood of breast cancer patients retained significantly higher levels of Trx1 than did that of other cancer patients. The mean value of serum Trx1 levels was detected at 40.12 ± 6.816 ng/mL in the breast cancer group (BC), with about a 40.2% increase compared to the normal female control. With reference to the serum level of Trx1 according to sub-type of breast cancer, it was 40.03 ± 6.793 ng/mL in lobular carcinoma of breast (BCL) and 40.22 ± 6.878 ng/mL in ductal carcinoma of breast (BCD). The serum Trx1 level in lobular carcinoma of breast was nearly same as that in ductal carcinoma of breast.

The Trx1 level in lung (LC), colorectal (CRC), and kidney (KC) were detected at 37.71 ± 8.254, 32.92 ± 7.864, and 31.12 ± 7.541 ng/mL, with about a 13.5%, 17.9%, and 10.7% increase, respectively, compared to the female/male normal control (NFM). The differences among the levels of LC, CRC, and KC are not statistically significant (P > 0.05), but the differences between BC and other cancers are significant (P < 0.0001).

In addition, measurements of serum Trx1 levels in the female normal control (n = 50) and the breast (n = 197) and other cancer groups (n = 111 for non-small cell lung cancer, n = 64 for CRC, and n = 30 for KC) were subjected to ROC curve analysis, and the results are summarized in Table 3.

**Table 3:** Parameters from ROC analysis on serum Trx1 levels in patients with various cancers

**Type of cancer**	AUC* (±SEM)	**Sensitivity (%)**	**Specificity (%)**	**Criterion (ng/ml)**	**# of serum (Grade)**
**Breast/NFM**	0.911 ± 0.179	89.3	78.0	>32.3390	197 (I/II/III)
**Colorectal/NFM**	0.687 ± 0.0425	68.3	61.0	>29.0533	64 (I/II/III)
**Kidney/NFM**	0.633 ± 0.0617	46.7	81.0	>32.9986	30 (I/II)
**Lung/NFM**	0.643 ± 0.0381	54.1	73.0	>31.1820	108 (I/II/III/IV)
**Normal Control**	-	-	-	-	100 F/M: 50 F / 50 M

*P < 0.0001.

As can be seen in Table 3, when compared to the female normal control (NF, n = 50) and the male normal control (NM, n = 50), the AUC (area under curve) value was measured at 0.911 ± 0.0179, and the cut-off value at >32.3390 ng/mL, with a sensitivity of 89.3% and a specificity of 78.0%.

Taken together with the parameters from ROC analysis on the serum Trx1 levels of various cancer patients summarized in Table 3, this data indicates that Trx1 can be used as a breast cancer marker capable of discriminating breast cancer patients from the female control at higher probability with superior sensitivity and specificity.

### Analysis of Trx1 as breast cancer-specific marker

To examine the selectivity of the blood marker Trx1 for breast cancer, the data of serum Trx1 levels for breast cancer in comparison to other cancers of shown in Figure 2 (lung cancer (LC), kidney cancer (KC) and colorectal cancer (CRC) was subjected to ROC curve analysis, and the results are shown in Table 4.

**Table 4:** Parameters from ROC analysis on serum Trx1 levels between patients with breast and other cancers

**Type of cancer**	AUC* (±SEM)	**Sensitivity (%)**	**Specificity (%)**	**Criterion (ng/ml)**
**BC/NMF**	0.911 ± 0.0179	89.3	78.0	>32.3390
**BC/CRC**	0.765 ± 0.0388	88.8	58.7	>33.5024
**BC/KC**	0.809 ± 0.0461	75.6	76.7	>35.3212
**BC/LC**	0.773 ± 0.0281	89.3	55.0	>32.2963

*P < 0.0001.

As is understood from the data of Figure 2, Tables 3 and 4, serum Trx1 levels were significantly higher in the breast cancer group than in other cancer patient groups as well as in the female and male normal control (NFM). In addition, when the serum Trx1 level was measured in breast cancer patients in comparison with other cancer patients, the AUC (area under curve) value exceeded about 0.75, with the sensitivity and selectivity detected at more than 75% and 55%, respectively, in all cases, indicating that Trx1 is useful as a breast cancer-specific marker.

### Sensitivity of Trx1 with the progress of cancer

**Figure 3:**
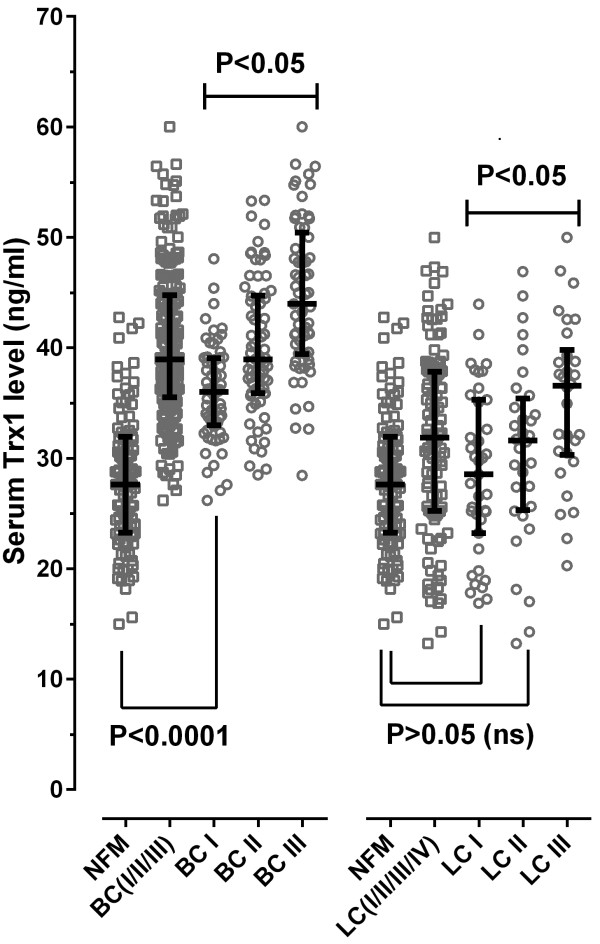
**Changes in serum Trx1 levels in patients with breast and lung cancers as a function of the progress of the cancers.** The individual mean value (n = 3) was depicted as a scatter dot plot with the median value of each group (horizontal lines) and the interquartile range (vertical lines). *Abbreviations*: BC, breast cancer patients; LC, lung cancer patients; I, II, III; divided corresponding cancer (grade I, II, and III, respectively).

In order to reexamine the selectivity of Trx1 as a breast cancer-specific marker and to confirm the proportional correlation of serum Trx1 level with the progress of breast cancer, a comparison was made with blood samples taken from many lung cancer patients (LC). The results are shown in Figure 3.

As shown in Figure 3, the serum Trx1 level exhibited a correlation with the progress of breast cancer in a pattern similar to that shown in the progress of lung cancer.

These data indicate that the increase of serum Trx1 level with the progress of cancer is due to the oxidative stress increased with the progress of cancer. However, the serum Trx1 level in the lung cancer group was about 21% lower than that in the breast cancer group, suggesting that the high increase in the breast cancer group is attributed to the specificity of Trx1 for breast cancer.

### Comparison of Trx1 with carcinoembryonic antigen (CEA) as a breast cancer marker

It was found that serum from individuals with various carcinomas including breast carcinoma had higher levels of CEA than healthy individuals. CEA is frequently used as a breast cancer marker. Therefore, we investigated the diagnostic capacity of Trx1 and CEA for the detection of breast cancer comparing the parameters from ROC curve analysis.

**Figure 4:**
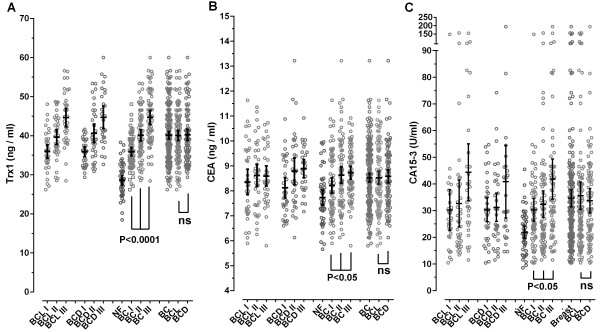
**Changes of serum Trx1, CEA, and CA15-3 levels in patients breast cancer as a function of the progress of the cancer.** The individual mean value (n = 3) was depicted as a scatter dot plot. The mean value of each group is shown by horizontal lines, and the 95% of confidence interval (CI) is displayed by vertical lines. *Abbreviations*: BCL, lobular carcinoma of breast; BCD; ductal carcinoma of breast; NF, normal female; I, II, III; grade 1, II, III of breast cancer, corresponding cancer grades, respectively. The average data of Trx1, CEA, and CA15-3 levels in sera were displayed in panels **A**, **B**, and **C**, respectively.

As depicted in Figure 4, increased serum levels of Trx1 and CEA were detected in breast cancer patients, but the increase of CEA levels (10%, from 7.731 ± 1.051 to 8.525 ± 1.282 ng/mL: mean ± SD) was significantly lower than that of Trx1 levels (56.8%, from 25.59 ± 4.211 to 40.12 ± 6.816 ng/mL: mean ± SD) compared to their values of corresponding female normal controls. Further, the both serum levels were observed to have a proportional correlation with the progress of breast cancer, with statistical significance, however the serum Trx1 level exhibited a superior correlation with the progress of breast cancer (P < 0.0001) compared to CEA (P < 0.05).

To examine the superiority of Trx1 as a blood marker for breast cancer, the data of serum Trx1 and CEA levels for breast cancer were subjected to ROC curve analysis, and the results are shown in Table 5. As shown in Table 5, the AUC value for Trx1 exceeded about 0.83, with the sensitivity and selectivity detected at more than 82% and 72%, respectively, in all the cancer grades from I to III. In case of CEA, as shown in Table 4, when compared to the female normal control (NF, n = 50), the AUC value was measured at 0.678 ± 0.0408, and the cut-off value at >8.28 ng/ml, with a sensitivity of 54.4% and a specificity of 77.6%. Taken together, the superiority as a breast cancer marker, compared to CEA, is attributed to the higher sensitivity of Trx1 than that of CEA.

**Table 5:** Parameters from ROC analysis on serum Trx1, CEA, and CA15-3 levels in patients with breast cancer

**BC Grade**	AUC* (±SEM)	**Sensitivity (%)**	**Specificity (%)**	Criterion** Value	**# of serum (# of L:-D) §**
**BC_Total**^**╔**^	**0.901 ± 0.0252**	**89.8**	**78.0**	**>32.1725**	197 (106:91)
	0.678 ± 0.0408	54.4	77.6	>8.28	
	0.179 ± 0.0359	48.6	89.8	>29.8227	
**BC I**	**0.837 ± 0.0399**	**89.4**	**72.0**	**-**	66 (35:31)
	0.594 ± 0.0523	30.3	89.8	-	
	0.693 ± 0.0482	50.0	83.7	-	
**BC II**	**0.908 ± 0.0270**	**82.9**	**86.0**	**-**	70 (37:33)
	0.692 ± 0.0478	56.8	77.6	-	
	0.673 ± 0.0480	44.8	89.8	-	
**BC III**	**0.962 ± 0.0156**	**88.5**	**92.0**	**-**	61 (34:27)
	0.762 ± 0.0460	66.7	77.6	-	
	0.794 ± 0.0411	60.6	89.8	-	
**Normal**	-	-	-	-	50 (NF)

*P < 0.0001.

**Units of criterion values represent as ng/ml for Trx1 and CEA or U/ml for CA15-3.

^╔^Upper, middle, and lower values in each row are those for Trx1, CEA, and CA 15–3, respectively.

§L and D are lobular and ductal carcinoma of breast cancer respectively.

Further, the AUC value for CEA in grade I breast carcinoma was measured at 0.594 ± 0.0523 with a sensitivity of 30.3% and a specificity of 89.8%. The AUC value for Trx1 even in grade I breast carcinoma (0.837 ± 0.0399) was higher than those for CEA in the all cancer grades (I-III). It is worth noting that the sensitivity for Trx1 (89.4%) is higher than that for CEA (30.3%), whereas the specificity for Trx1 (72.0%) is lower than that for CEA (89.8%). Therefore, a combination of the serum Trx1 and CEA in patients with breast carcinoma can increase their diagnostic capacity for the early detection of breast cancer.

**Figure 5:**
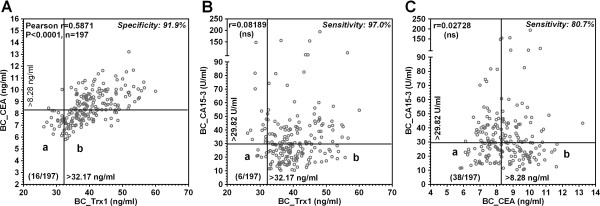
**Correlation between serum markers for breast cancer. A** denotes the correlation between the serum Trx1 and CEA levels in patients with breast cancer (BC); **B**, the correlation between the serum Trx1 and CA15-3 levels in breast cancerous females; **C**, the correlation between the serum CEA and CA15-3 levels in patients with breast cancer. Vertical and horizontal solid lines indicate the cut-off values for corresponding marker for breast cancer. The individual values are the average values of three (for Trx1 and CEA) or two (for CA15-3) measurements.

As depicted in Figure 4, both serum levels of Trx1 and CEA in breast cancer are elevated as a function of the progress of breast carcinoma. We plotted the individual serum levels of Trx1 in 197 breast cancer serums along the x-axis and that of CEA in the corresponding sample along the y-axis. Figure 5A displays a good positive correlation between the levels of patients (Pearson r = 0.58971; P < 0.0001), respectively, suggesting Trx1 sustains the serum of patients with breast cancer in accordance with CEA. The false negative group within the box (region b in Figure 5A) determined by the cut-off value of CEA (8.28 ng/ml) are rescued from wrong diagnosis by diagnosing breast cancer with the cut-off value of Trx1 (32.17 ng/ml), which is due to the superior sensitivity of Trx1 (89.8%) compared to that of CEA (54.4%). The false negative patients determined by both cut-off values were depicted in region a (16 patients of 197 total patients: 8.1%).

### Comparison of Trx1 with CA15-3 as a breast cancer marker

It was found that serum from individuals with various carcinomas, including breast carcinoma, has higher levels of CA15-3 than does serum from healthy individuals. CA15-3 is frequently used as a breast cancer marker, but it by itself is not an effective screening method for early-stage breast cancer, mainly due to its lack of sensitivity [22]. Therefore, we comparatively investigated the diagnostic capacities of Trx1 and CA15-3 for the detection of breast cancer and compared their parameters from respective ROC curve analysis.

As shown in Figure 4B, increased serum level of CA15-3 was detected in breast cancer patient (∼58%, from 21.92 ± 7.854 U/ml to 34.68 ± 24.14 U/ml; mean ± SD). Serum CA15-3 levels were observed to have a proportional correlation with the progress of breast cancer, but they exhibited a less correlation with the progress of breast cancer (P < 0.05, from one-way ANOVA analysis) compared with Trx1 (P < 0.0001).

To compare the utility of CA15-3 and Trx1 as blood markers for breast cancer, the serum CA15-3 levels for breast cancer were subjected to ROC curve analysis, and the results are shown in Table 5. For CA15-3, when compared to the female normal control, the AUC value was measured at 0.719 ± 0.0359 with a sensitivity of 48.6% and a specificity of 89.8%. Further, the AUC value for CA15-3 in the early-stage (stage I) breast carcinoma was measured at 0.693 ± 0.0482 with a sensitivity of 50.0% and a specificity of 83.7%. The AUC value for Trx1 even in the stage I breast carcinoma (0.837 ± 0.0399) was higher than that for CA15-3 in the corresponding cancer stage (0.691 ± 0.0482). The sensitivity for Trx1 (89.8%) is much higher than that for CA15-3 (48.6%), whereas the specificity for Trx1 (78.0%) is lower than that for CA15-3 (89.8%). Therefore, a combination of the serum Trx1 and CA15-3 levels in patients with breast cancer could improve the diagnostic sensitivity of CA15-3 for detection of breast cancer.

As depicted in Figure 4A and 4C, both serum levels of Trx1 and CA15-3 in breast cancer are elevated compared to corresponding control. We plotted the levels of Trx1 in 197 individual breast cancer serums along the x-axis and of CA15-3 in the corresponding sample along the y-axis. Figure 5B shows no correlation between the patients (Pearson r = 0.08189; P = 0.2526), suggesting Trx1 is sustained in the serum of patients with breast cancer in a quite different manner from CA15-3 and also supporting Trx1 as an complementary and thus effective companion marker to CA15-3. The false negative patients within the box (region b in Figure 5B) determined by the cut-off value of CA15-3 (29.8227 U/mL) are rescued from the wrong diagnosis by diagnosing breast cancer with the cut-off value of Trx1 (32.1725 ng/mL), due to the higher sensitivity of Trx1 (89.8%) compared to CA15-3 (48.6%). The false negative patients determined by both cut-off values were depicted in region a (6 patients of 197 total patients: 3%).

The false negative patients (38 patients of 197 total patients; 19.3%) determined by both cut-off values of CA15-3 (29.8227 U/mL) and CEA (8.28 ng/mL) were depicted in region “a” shown in Figure 5C. Figure 5C showed no correlation between the patients (Pearson r = 0.02728; P = 0.7029), suggesting both markers are sustained in the serum of patients with breast cancer in quite different manners and suggesting the combined use of CEA with CA15-3 is effective for screening the patients with breast cancer.

Taken together, we suggest that the Trx1 blood test itself is reliable for diagnosing breast cancer or as a screening test for early detection of breast cancer, and moreover that the combinational use of Trx1 with either CEA or CA15-3 overcomes their problems (poor sensitivity) encountered in diagnosis by the CEA or CA15-3 test alone.

### Relationship of serum Trx1 levels in patients with breast cancer with age and status of menopause

**Figure 6:**
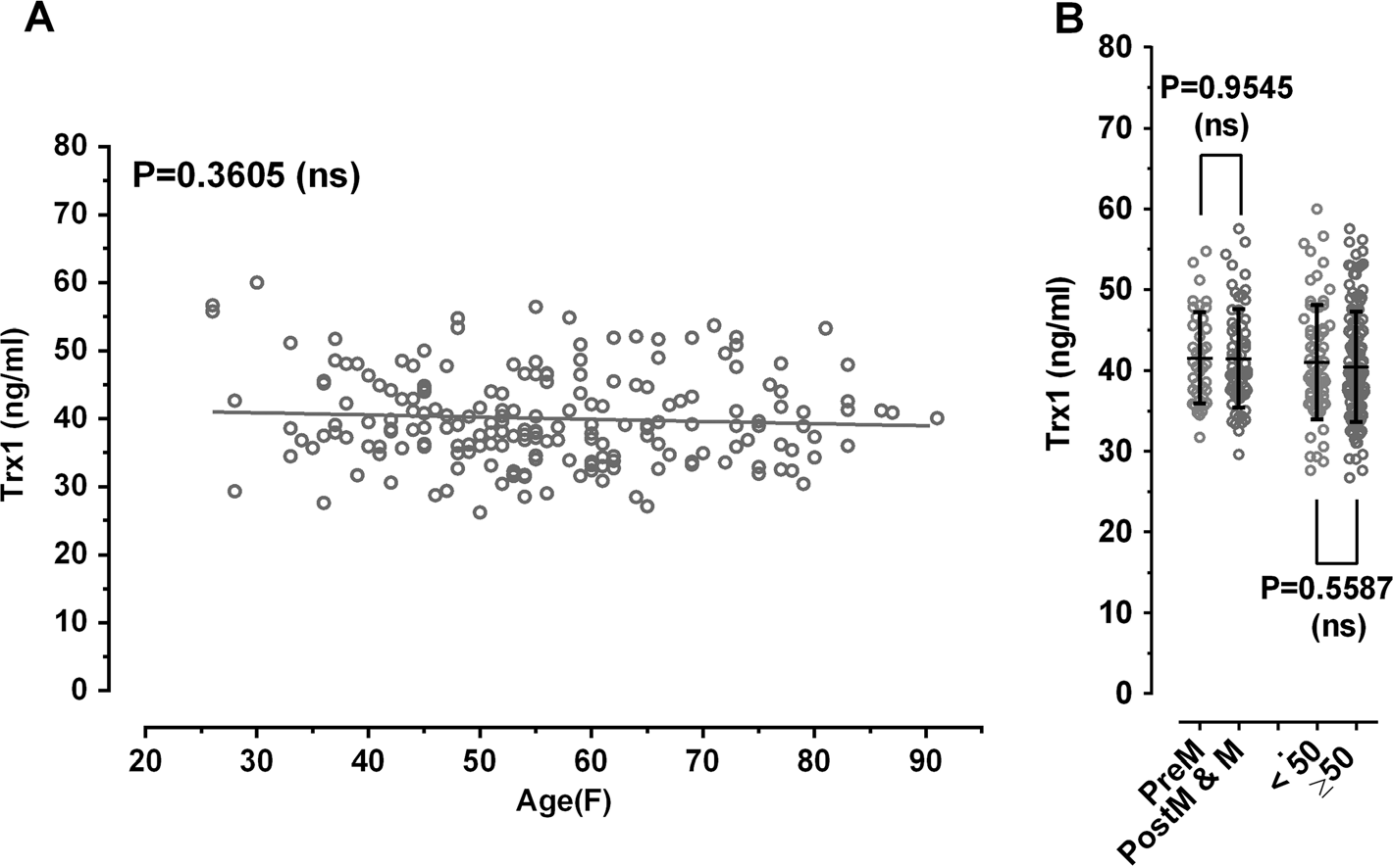
**Relationship of serum Trx1 levels in patients with breast cancer with age and status of menopause.** Clinical information for patients with breast cancer was provided by the supplier. The individual values are the average values of three measurements. Panel **A** denotes no relationship between serum level of Trx1 and age of breast cancer patient. Panel **B** shows no relationship between serum level of Trx1 and menopause status of patient with breast cancer.

The measurement of serum levels of Trx1 was performed on the sera of 197 breast cancer patients over a wide range of ages with a uniform distribution (48.5 ± 18.43; mean ± SD). The individual data were depicted as a function of age. As shown in Figure 6A, the serum levels appear to be slightly decreased as the function of age (slope = −0.032 ± 0.0348), but they showed no statistical significance (P = 0.3605).

As shown in Figure 6B, the Trx1 level in the patients over 50 (40.44 ± 6.791 for age ≥50; mean ± SD) was not significantly different from that in the patients below 50 (41.05 ± 7.078 for age <50) (P = 0.5587). The level in the menopausal and post-menopausal patients (41.49 ± 6.0171, n = 66) was not significantly different from that in the pre-menopausal patients (41.55 ± 5.610, n = 40) (P = 0.9545).

## Discussion

In the previous study, the expression levels of Trx1 in human normal tissue and cancerous tissue were examined [21]. Trx1 was found to be expressed at the lowest level in normal breast tissue among 48 different normal human tissues, and at a higher level in breast cancer tissue than in other cancerous tissue, as measured by qRT-PCR and Western blotting. In addition, the more progressed the cancer is, the higher the induction fold of mRNA expression of Trx1. Thus, the induction fold of mRNA expression of Trx1 becomes high in stage II-IV breast cancer, particularly stage IV breast cancer, that is, metastatic breast cancer. Further, the induction fold of mRNA expression of Trx1 is closely associated with the malignancy of cancer as it increases with the progression of cancer. Therefore, the induction fold of mRNA expression of Trx1 is associated with subdivision of cancer stages. Found to be overexpressed in human breast cancer tissues, as described above, Trx1 allows the diagnosis and prognosis of breast cancer and thus is useful as a diagnostic marker for breast cancer.

The present study provides a diagnostic marker for breast cancer, comprising the Trx1 level in blood. Breast cancer patients were found to have significantly higher serum Trx1 levels than normal persons, as measured by an indirect ELISA. In addition, comparison between breast cancer and other cancers showed that significantly high Trx1 levels were detected in blood taken from breast cancer patients, compared to patients with other cancers. Moreover, the serum Trx1 level exhibited a proportional correlation with the progress of breast cancer. Analysis of serum Trx1 level in normal female and patients with breast cancer as function of age revels that the level in female control seems to increase as a function of age (P = 0.0848) (Figure 1B) indicating a weaker association with the age, but the patient level does not response to increase of age (P = 0.3605) (Figure 6A). The slight increase of serum Trx1 level in normal female in older female control could be due to the oxidative stress increased with age. No relationship of Trx1 levels in the patients with their ages suggests that significant elevation of the trx1 levels would reflect the specificity of Trx1 as a breast cancer marker.

We also analyzed data from the pre-menopausal and post-menopausal patients. As shown in Figure 6B, the status of menopause is not associated with the level of Trx1 in patients with breast cancer (P = 0.9545). Taken together, these results indicate that there are no significant relationships between the Trx1 level and menopause status of the patients. Based on the observation, we suggest that age and menopausal status do not act as considerable factor to affect serum Trx1 level regardless of the presence or absence of breast cancer although age and menopausal status are well established risk factor for breast cancer [22].

Carcinoembryonic antigen (CEA) was first identified in 1965 by Phil Gold and Samuel O. Freedman in human colon cancer tissue extracts [23]. CEA is a glycoprotein involved in cell adhesion [24]. It is normally produced during fetal development, but the production of CEA stops before birth. Therefore, it is not usually present in the blood of healthy adults, although levels are raised in heavy smokers. It was found that serum from individuals with colorectal carcinoma, gastric carcinoma, pancreatic carcinoma, lung carcinoma and breast carcinoma, as well as individuals with medullary thyroid carcinoma, had higher levels of CEA than healthy individuals [25-27]. Analysis of detection of CEA in breast cancer patients leads to that the CEA blood test is not reliable for diagnosing breast cancer or as a screening test for early detection of the cancer especially due to the lack of sensitivity (54.4% for all grades of the cancer from I to III, 30.3% for the grade I) (Figure 4B and Table 5). If the test is not *sensitive*, then it may miss cancers. Therefore, the CEA breast cancer screening tests miss a large number of patients with breast cancer depicted in Figure 5B. This wrong diagnosis becomes severer in the early-phased patients (grade I) with a sensitivity of 30.3% (Table 5).

Cancer antigen (CA) 15–3 is a circulating MUC-1 antigen in peripheral blood that is a normal product of breast tissue, and it does not cause breast cancer. One of the widely used tumor marker in breast cancer is CA15-3, but it is not sensitive or specific enough to be considered useful as a tool for cancer screening. Its main use is to monitor a person's response to breast cancer treatment in case of the cancer that overproduces CA15-3 and to help watch for breast cancer recurrence. The present data also indicate that the CA15-3 blood test on breast cancer patients is not reliable for diagnosing breast cancer or as a screening test for early detection of the cancer due to its lack of sensitivity. The breast cancer screening test by CA15-3 misses a large number of breast cancer patients. This wrong diagnosis becomes more severe in the early-phase patients, with a sensitivity of only 50.0% and 44.8% for the stage I and stage II, respectively (Table 5).

In breast cancer as well as in other solid tumors all tumor markers actually available are not tumor specific. There are some reports concerning serum breast cancer markers such as CEA, and CA15-3, and class I alcohol dehydrogenase (ADH). Among all tested classes of ADH isoforms, only class I had higher activity in the serum of patients with breast cancer in stage IV [28,29]. However, most of breast cancer markers show lack of the diagnostic sensitivity and specificity.

In a combination analysis we investigated the diagnostic capacity of Trx1 and CEA for the detection of breast cancer comparing the use of fixed cut off values. On this combination analysis we reached a sensitivity of 90.9% for both markers (Figure 5A). Considering the sensitivity (54.4%) in all stages of breast cancer for CEA alone, this combination analysis with Trx1 is helpful to rescue 36.5% of patients with breast cancer from wrong diagnosis.

In another combined analysis, we investigated the diagnostic capacity of Trx1 and CA15-3 for the early detection of breast cancer, comparing the use of fixed cut-off values. In this combination analysis, shown in Figure 5B, we reached a sensitivity of 97.0% for both markers. Considering the sensitivity of CA15-3 (48.6%) alone, this combination analysis with Trx1 could help rescue 48.4% of patients with breast cancer from the wrong diagnosis.

Finally, we examined the diagnostic capacity of CEA and CA15-3 as the combinational markers for patients with breast cancer. This combination analysis also significantly increases sensitivity for both markers compared to the individual sensitivity for CEA and CA15-3 (a sensitivity of 48.6% for CEA alone, 48.6% for CA15-3 alone, and 80.7% for both markers) (Figure 5C).

In summary, considering the sensitivities derived from each of three sets of combinational analyses, the set of Trx1 and CA15-3 is the most efficient combination for the detection of the patients with breast cancer among three sets of combination (a sensitivity of 97.0% for a set of Trx1 and CA15-3, 91.9% for a set of Trx1 and CEA, and 80.7% for set of CEA and CA15-3). Comparison of Trx1 with CEA or CA15-3 as a serum breast cancer marker suggests that Trx1 is the most superior marker for breast cancer.

## Conclusions

Comparison of breast cancer with other cancers showed that the highest Trx1 levels with its high sensitivity and specificity were detected in blood taken from breast cancer patients, compared to patients with other cancers and normal persons. Moreover, the serum Trx1 level exhibited a proportional correlation with the progress of breast cancer. Taken together, we conclude that serum Trx1 is the most useful marker for the prognosis and early diagnosis of breast cancer. In addition, we suggest that the diagnostic capacity of CEA or CA15-3 alone for the early detection of breast cancer, especially regarding sensitivity, would be significantly improved by its combination with Trx1.

## Abbreviations

ROS: Reactive oxygen species; Trx: Thioredoxin; CEA: Carcinoembryonic antigen; CA15-3: Cancer antigen 15–3; ROC: The receiver operating characteristic curve; AUC: Area under curve.

## Competing interests

The authors declare that they have no competing interests.

## Authors’ contributions

IHK conducted the work, analyzed the data and wrote the manuscript. MKC and BJP performed the experiments throughout this work. All authors have read and approved the final manuscript.
